# Glutamate-Induced Deregulation of Krebs Cycle in Mitochondrial Encephalopathy Lactic Acidosis Syndrome Stroke-Like Episodes (MELAS) Syndrome Is Alleviated by Ketone Body Exposure

**DOI:** 10.3390/biomedicines10071665

**Published:** 2022-07-11

**Authors:** Sophie Belal, David Goudenège, Cinzia Bocca, Florent Dumont, Juan Manuel Chao De La Barca, Valérie Desquiret-Dumas, Naïg Gueguen, Guillaume Geffroy, Rayane Benyahia, Selma Kane, Salim Khiati, Céline Bris, Tamas Aranyi, Daniel Stockholm, Aurore Inisan, Aurélie Renaud, Magalie Barth, Gilles Simard, Pascal Reynier, Franck Letournel, Guy Lenaers, Dominique Bonneau, Arnaud Chevrollier, Vincent Procaccio

**Affiliations:** 1 MitoLab Team, UMR CNRS 6015-INSERM U1083, Unité MitoVasc, SFR ICAT, Université d’Angers, 49933 Angers, France; sophie.belal@gmail.com (S.B.); dgoudenege@sb-roscoff.fr (D.G.); cinzia.bocca@univ-angers.fr (C.B.); jmchaodelabarca@chu-angers.fr (J.M.C.D.L.B.); vadesquiret@chu-angers.fr (V.D.-D.); nagueguen@chu-angers.fr (N.G.); ge.geffroy@gmail.com (G.G.); rayane.school@gmail.com (R.B.); selmakane@hotmail.com (S.K.); salim.khiati@univ-angers.fr (S.K.); cebris@chu-angers.fr (C.B.); a-inisan@hotmail.fr (A.I.); aurelie.renaud@univ-angers.fr (A.R.); pareynier@chu-angers.fr (P.R.); guy.lenaers@univ-angers.fr (G.L.); dobonneau@chu-angers.fr (D.B.); arnaud.chevrollier@univ-angers.fr (A.C.); 2 Biochemistry and Genetics Department, University Hospital of Angers, 49933 Angers, France; mabarth@chu-angers.fr (M.B.); gisimard@chu-angers.fr (G.S.); 3 Signalling and Cardiovascular Pathophysiology, INSERM UMR-S 1180, University of Paris-Saclay, 92296 Châtenay-Malabry, France; florent.dumont@u-psud.fr; 4 Institute of Enzymology, Research Center for Natural Sciences, H-1519 Budapest, Hungary; aranyi.tamas@ttk.hu; 5 Department of Molecular Biology, Semmelweis University of Medicine, H-1519 Budapest, Hungary; 6 Ecole Pratique des Hautes Etudes, PSL Research University, 75014 Paris, France; daniel.stockholm@ephe.sorbonne.fr; 7 Centre de Recherche Saint-Antoine, UMRS-938, INSERM, Sorbonne Université, F-75012 Paris, France; 8 Department of Neurobiology-Neuropathology, Angers Hospital, 49933 Angers, France; franck.letournel@univ-angers.fr; 9 UMR INSERM 1066-CNRS 6021, MINT Laboratory, 49933 Angers, France; 10 Service de Neurologie, CHU d'Angers, 49933 Angers, France

**Keywords:** mitochondrial diseases, mtDNA, MELAS syndrome, multi-omics, glutamate, tricarboxylic acid cycle, NADH/NAD imbalance, ketone body treatment

## Abstract

(1) Background: The development of mitochondrial medicine has been severely impeded by a lack of effective therapies. (2) Methods: To better understand Mitochondrial Encephalopathy Lactic Acidosis Syndrome Stroke-like episodes (MELAS) syndrome, neuronal cybrid cells carrying different mutation loads of the m.3243A > G mitochondrial DNA variant were analysed using a multi-omic approach. (3) Results: Specific metabolomic signatures revealed that the glutamate pathway was significantly increased in MELAS cells with a direct correlation between glutamate concentration and the m.3243A > G heteroplasmy level. Transcriptomic analysis in mutant cells further revealed alterations in specific gene clusters, including those of the glutamate, gamma-aminobutyric acid pathways, and tricarboxylic acid (TCA) cycle. These results were supported by post-mortem brain tissue analysis from a MELAS patient, confirming the glutamate dysregulation. Exposure of MELAS cells to ketone bodies significantly reduced the glutamate level and improved mitochondrial functions, reducing the accumulation of several intermediate metabolites of the TCA cycle and alleviating the NADH-redox imbalance. (4) Conclusions: Thus, a multi-omic integrated approach to MELAS cells revealed glutamate as a promising disease biomarker, while also indicating that a ketogenic diet should be tested in MELAS patients.

## 1. Introduction

Mitochondria, as the power plant of the cells, produce ATP through the oxidative phosphorylation (OXPHOS) process that takes place in the inner mitochondrial membrane [1]. Mitochondrial diseases arise from pathogenic variants in mitochondrial DNA (mtDNA) or in nuclear genes that encode proteins essential for mitochondrial functions [2,3]. The mitochondrial genome encodes 22 transfer RNAs (tRNAs) and 2 ribosomal RNAs (rRNAs) required for the translation of 13 polypeptides, all included in the OXPHOS complexes [1].

One of the most commonly encountered mtDNA mutations involved in mitochondrial disorders is the m.3243A > G pathogenic variant in *MT-TL1*, which is responsible for the life-threatening MELAS syndrome [4] when present in high mutation loads. Although clinically heterogeneous, MELAS syndrome is characterised by a triad of symptoms including lactic acidosis, stroke-like episodes before the age of 40, and encephalopathy [5]. MELAS syndrome also induces a wide spectrum of additional symptoms such as epilepsy, diabetes, exercise intolerance, myopathy, dementia, and deafness [6].

The severity of MELAS syndrome is related to the m.3243A > G heteroplasmy level, reflecting the coexistence of wild type and mutant mtDNA copies [1,3]. The m.3243A > G variant affects the *MT-TL1* gene, encoding the tRNA^Leu(UUR)^ which leads to the alteration of its structure, stability, aminoacylation, and codon recognition, thus reducing the efficiency of the mitochondrial translational machinery [7,8,9]. MELAS is associated with multiple partial respiratory chain defects, particularly involving mitochondrial complex I and/or complex IV deficiencies [4,10,11,12]. However, the complex I deficiency is often viewed as the key element of the disease [13,14,15]. Additional rare pathogenic variants responsible for MELAS have been also identified in other genes that encode mitochondrial tRNA, affecting either the synthesis of proteins or NADH dehydrogenase (ND) subunits (i.e., ND1, ND5, and ND6 subunits). These variants have been functionally validated by the reduction of complex I activity in neuronal cellular models of MELAS [16,17], further highlighting the crucial role of complex I deficiency in the pathophysiology of the disease [5,18].

The considerable efforts made to better understand the pathophysiology of MELAS have so far not led to the discovery of specific effective therapies [1,11]. 

Several compounds have been used in individuals affected by MELAS including coenzyme Q10, L-arginine, and citrulline, but none of them have shown real clinical efficacy [1,10,11], particularly in alleviating stroke-like episodes [19]. Alternatively, a ketogenic diet, which combines carbohydrate restriction and ketone body (KB) supplementation has been used for decades to treat drug-resistant epilepsy [20]. This success has prompted testing of the ketogenic diet to treat other severe neurological disorders, including MELAS syndrome [21,22,23]. It has previously been shown that long-term exposure to KB significantly improves complex I activity and assembly [21] in MELAS neuronal cybrids, where there is a strong reduction in mitochondrial complex I activity and assembly identical to what is observed in fibroblasts and muscle biopsies obtained from MELAS patients [13,16,17,24].

In order to thoroughly assess the consequences of the m.3243A > G MELAS variant, we used a multi-omic approach on cybrid neuronal cells with the MELAS mutation to unravel the metabolic pathways and identify potential biomarkers. We then tested the effects of exposure to KB on the mitochondrial dysregulation observed. 

## 2. Materials and Methods

### 2.1. Cell Culture

The SH-SY5Y neuronal control cells and mutant cybrid cells carrying m.3243A > G with 98%, 90%, and 70% mutation loads and 143B osteosarcoma cybrid control and mutant cells were cultured in standard DMEM high glucose media (4.5 g/L) or in low glucose (0.5 g/L) (PAN biotech, Aidenbach, Germany), supplemented with 10% foetal bovine serum (PAN biotech), 1% glutamine, and 50 μg/mL uridine (Sigma-Aldrich, Saint-Louis, MO, USA) at 37 °C in the presence of 5% CO_2_ as described elsewhere [16]. Control and mutant cells were exposed to 5 mM of acetoacetate and β-D-hydroxybutyrate (Sigma-Aldrich, Saint-Louis, MI, USA) in a low glucose medium for 48 h. Control cells were exposed to rotenone (200 nM) a complex I inhibitor for 15 h.

### 2.2. Deconvolution Microscopy

To assess the mitochondrial network, cells were incubated for 20 min with MitoTracker green FM (Invitrogen™, Carlsbad, CA, USA) and 5 min 0.1 ug/mL Hoechst 33342 to visualise the mitochondria (green) and nucleus (blue). Coverslips were analysed using a microscope ECLIPSE Ti-E equipped with a Plan Apo100x N.A. 1.45 oil immersion objective (Nikon, Tokyo, Japan).). To analyse the mitochondrial network, images acquired by using an Andor NEO sCOMS camera controlled by Metamorph 7.7 software (Molecular Devices) were deconvolved (Huygens Essential, 14.06 version Scientific Volume Imaging Hilversum, The Netherlands) and quantified (Imaris 8.0 ^®^ software (Oxford Instruments Brand Bitplane AG, Zurich, Switzerland). 

### 2.3. Mitochondrial Enzyme Activities 

Activities of complexes I, II, and SDH (succinate dehydrogenase) of the respiratory chain were determined at 37 °C with an UV*mc*^2^ spectrophotometer (SAFAS, Monaco) on mitochondrial fractions [25].

### 2.4. Mitochondrial Respiration Measurements 

Oxygen consumption was quantified in non-permeabilised cells using oxygraphic equipment (Oroboros, Innsbruck, Austria). The O_2_ consumption was determined in routine respiration and after ATP synthase inhibition with oligomycin (1 mg/mL). The maximal respiration capacity was then measured by the addition of increasing doses of uncoupler FCCP up to the maximal respiration rate [17].

### 2.5. Targeted Metabolomic Analysis of Cell Homogenates 

Biocrates AbsoluteIDQ p180 kit (Biocrates Life sciences AG, Austria) was used for a targeted quantitative metabolomic approach (Biocrates Kit User Manual). The mass spectrometer AB Sciex QTRAP 5500 (Life Sciences SCIEX, Framingham, MA, USA) was able to quantify up to 188 different endogenous molecules, among them acylcarnitines, amino acids, biogenic amines, glycerophospholipids, sphingolipids, and sugar as described elsewhere [26]. The quantification of acylcarnitines, glycerophospholipids, sphingolipids, and sugar was performed with Flow-injection analysis (FIA-MS/MS). The separation of amino acids and biogenic amines has been performed with liquid chromatography (LC) prior to detection with mass spectrometry (LC-MS/MS). 

Detection of TCA intermediates using non-targeted metabolomics. In order to complete the targeted signature obtained with the Biocrates Absolute IDQ p180 kit, the non-targeted reversed-phase (RP) metabolomics approach that had been previously validated and published was adapted [27]. The cell layers were rinsed two times with an aqueous solution of 0.22% NaCl before being washed with cold MeOH. The cell suspension was then aliquoted into one million cell pellets and stored at −80 °C until analysis. Internal quality controls (QC) were obtained by mixing the samples before the extraction protocol and processed in a similar manner. Krebs cycle intermediates were then extracted by adding a mixture of H_2_O/MeOH. Supernatants were evaporated after one centrifugation. Samples were reconstituted with an aqueous solution (2% MeOH) and analysed in negative mode Ultra High Pressure Liquid Chromatography-High Resolution Mass Spectrometry on a Q Exactive mass spectrometer (Thermo Fisher Scientific GmbH, Bremen, Germany) coupled to UltiMate^®^ 3000 UHPLC (Dionex Softron GmbH, Germering Germany). The ionisation, chromatography, and MS parameters can be found in the following references [27,28,29]. The extracted data was then processed by using Simca P 16.0 (Umetrics, Umea, Sweden). 

### 2.6. Metabolite Quantification

Glutamate intracellular or extracellular concentrations were quantified in line with the manufacturer’s recommendations (Glutamate Assay Kit, Abcam Amsterdam, Netherlands). Amino acid chromatography in post-mortem brain tissues was performed using a routine analytical procedure that can accurately detect and quantify 37 amino acids and amino acid-derived molecules by means of a UptiSphere BP2 chromatography column coupled with an API 3000 Triple Quadrupole Mass Spectrometer (AB Sciex^®^ Darmstadt, Germany). Frozen pellets of mutant cells were suspended in 100 µL specific lysis buffer (sucrose 250 mm, EGTA 0.5 mm, HEPES 10 mm, Triton 10X, and Antiprotease cocktail).

### 2.7. DNA Extraction and Quantification of mtDNA Heteroplasmy 

DNA extraction and quantification of the mutation load were performed as described elsewhere [16]. 

### 2.8. Gene Expression Profiling and Microarray Hybridisation

The TRIzol solubilization and extraction method was used for deproteinizing total RNA (Invitrogen Carlsbad, CA, USA.) and RNeasy Micro kit (Qiagen, Netherlands) for RNA preparation. RNA quantity and quality were assessed by Nanodrop 1000 (Thermo Fisher Scientific, Waltham, MA, USA) and Bioanalyzer 2100 together with an RNA6000 Nano kit (Agilent Technologies, Santa Clara, CA, USA). An RNA integrity number of 700 or more was achieved for all samples. Biotinylated, amplified cRNA was generated using the Illumina Total Prep RNA Amplification kit (Ambion, Life Technologies, Carlsbad, CA, USA). cRNA was hybridised on Illumina HumanHT-12 v4 Expression BeadChips, stained, and detected with the iScan system, in accordance with the manufacturer’s protocol (Illumina, CA, USA). GenomeStudio 2011 (version 1) and its Expression Analysis package (version 1.9.0) were used for signal retrieval and quantile normalisation (Illumina, CA, USA). Gene expression profile datasets are available from the following database: GEO accession number GSE165953. 

### 2.9. Quality Assessments, Normalisation, and Statistical Analysis of Gene Expression 

Data were analysed using R version 3.6.3 and Rstudio version 1.3.959. Raw data matrices were obtained from idat files using the Illumina package [30]. For the normalisation step, we applied a quantile normalisation using Limma with a log2 transformation to obtain a normalised data matrix [31,32]. As part of quality control, unsupervised analysis was scored using hierarchical clustering (Pearson’s dissimilarity and full linkage) and Principal Component Analysis (PCA). No technical bias or outlier sample had to be taken into account in the downstream analysis. To determine which genes were differentially expressed between control cells and cells with the MELAS mutation, we conducted a t-test on gene variables. Fold-changes were computed with delog values. Functional analysis was performed through the use of the MSigDB gene sets database [33] using binomial enrichment tests. The expression data are available through the NCBI Gene Expression Omnibus (GEO; accession number: GSE165953) on the GEO NCBI site.

### 2.10. Quantitative PCR

The RNeasy Mini Kit (Qiagen) was used for RNA purification. An amount of 2 µg of RNA was reverse-transcribed. Each 10 μL reaction of qRT-PCR contained 100 ng of cDNA, 5 μL of Power SYBR-Green PCR Master Mix (Applied Biosystems), and 0.5 μM of each forward and reverse primer and was performed in triplicates. Two sets of primers were developed to measure the mRNA expression levels of GAD67, ABAT, and GAPDH, and actin was used as control (primer sequences are available on request).

### 2.11. Western Blot Analysis

Protein preparation and GAD1, GDH, ABAT, Tubulin, and Actin (Abcam Amsterdam, Netherlands) quantifications were performed as described elsewhere [17]. 

### 2.12. Immunocytochemistry

Slices (5 mm thick) were cut from formalin-fixed, paraffin-embedded brain biopsy blocks using a Leica RM125 microtome. Immunohistochemistry was carried out using an automated slide immunostainer (Leica, Wetzlar, Germany). Incubation for 10 min with a rabbit monoclonal primary antibody that targeted GAD1, ABAT, or GDH was followed by an immunoperoxidase-based detection and a haematoxylin counterstain. Staining was examined microscopically and scored semi-quantitatively as follows: 0 = absent, + = mild, ++ = moderate, and +++ = strong.

### 2.13. Statistical Analysis 

Data are presented as the mean +/− SEM of at least 4 independent experiments. Differences between groups were evaluated by the Mann–Whitney statistical test using GraphPad Prism software (GraphPad Software, San Diego, CA, USA). The asterisk and the sharp sign (* and **#**) indicate *p*-value < 0.05, (** and ##) *p*-value < 0.001, and (***) *p*-value < 0.0001. 

### 2.14. Study Approval

Patient samples were obtained after informed consent was received (Institutional Review Board Committee of the University Hospital of Angers, Authorisation number: AC-2012-1507). Post-mortem brain tissue samples of an individual with MELAS, a woman who died at 32 years of age, and age-gender matched control brain samples were collected and frozen immediately after death.

## 3. Results

### 3.1. A Multi-Omic Approach Highlights Specific Signatures in Cells with the MELAS Mutation

**Figure 1:**
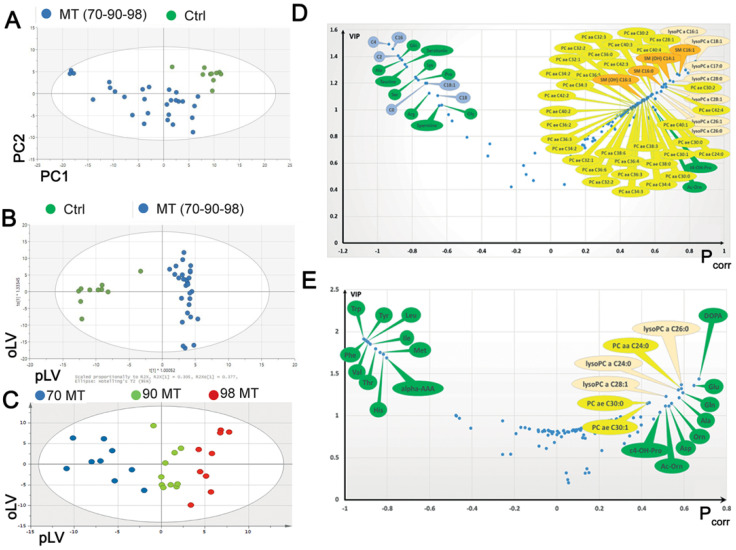
Metabolomic analysis between MELAS and control cells. (**A**) Unsupervised Principal Component Analysis (PCA) scatter plot carried on metabolomics data from parental control cells (n = 10, green dots) and mutant (MT, blue dots) cells with different m.3243A > G mutation loads (n = 30). The first two principal components (PC1 and PC2) explain more than 75% of the total variance. Control and mutant cells clearly group separately with control cells plotting in the upper right quadrant of the first principal plan determined by PC1 and PC2. (**B**) Supervised Orthogonal Partial Least Squares-Discriminant Analysis (OPLS-DA) scatter plot model aiming at classifying parental control cells from mutant cells based on the metabolomic data matrix. As expected from the PCA plot, both populations are very well separated in a predictive and non-overfitted model. Predictive (pLV) and orthogonal (oLV) latent variables are dimensionless. (**C**) OPLS score plot of MELAS cells with different MT loads. MT cybrids are separated according to the mutation load: 70% (blue circles), 90% (green circles), and 98% (red circles), along with the predictive latent variable (pLV). (**D**) Volcano plot obtained from the supervised OPLS models for MT cells with 70%, 90% to 98% mutation loads vs parental cells (Figure 1B). Only the most discriminating metabolites with high Variable importance on projection (VIP Ipiab and their loading rescaled as the correlation coefficient between the predictive latent variable and the corresponding metabolite or Pcorr (≥ 0.02 or ≤−0.02) have been labelled. Negative coefficients (left) indicate diminished metabolite concentrations in MT cells versus parental cells, whereas positive coefficients (right) indicate increased metabolite concentrations. (**E**) Volcano plot for the OPLS-DA model obtained from the metabolomic analysis of MELAS cells with 70%, 90%, and 98% mutation loads (Figure 1C). Only the most discriminating metabolites with high variable importance in the projection (VIP) values (>1) and Pcorr values (OPLS-DA model obtained from the metabolomic analysis of MELAS cells with 70%, 90%, 98% mutation loads (Figure 1C). Only the most discriminating metabolites with high variable importance in the projection (VIP) values (>1) and amino acids and biogenic amines are represented as green bubbles; phosphatidylcholines (PC) as yellow bubbles and lysophosphatidylcholines (lysoPC) as pink bubbles. In PC, “aa” indicates that both moieties at the sn-1 and sn-2 positions are fatty acids and bound to the glycerol backbone via ester bonds, whilst “ae” denotes that one of the moieties, either in the sn-1 or sn-2 position is a fatty alcohol and bound via an ether bond. For lysoPCs and PCs, the total number of carbon atoms and double bonds present in the lipid fatty acid chain(s) are denoted as “C x:y”, where x is the total carbon number (of both chains for PCs) and y is the total number of double bonds. Ala: Alanine; alpha-AAA: α-Aminoadipic acid; Ac-Orn: Acetylornithine; Asp: Aspartate; c4-OH-Pro: cis-4-Hydroxyproline; DOPA: 3,4-Dihydroxyphenylalanine; Gln: Glutamine; Glu: Glutamate; His: Histidine; Ile: Isoleucine; Leu: Leucine; Met: Methionine; Orn: Ornithine; Phe: Phenylalanine; Thr: Threonine; Trp: Tryptophane; Tyr: Tyrosine; Val: Valine. The metabolic signature is characterised by lower levels of 6 acylcarnitines (C0, C2, C4, C16, C18, C18:1) (blue bubbles), 10 amino acids and biogenic amines (green bubbles) and higher levels of several PC (yellow bubbles) and sphingomyelins (orange bubbles). Ala: Alanine, Gln: Glutamine, Ser: Serine, Lys: Lysine, Pro: Proline, Gly: Glycine, Arg: Arginine, Taurine, Serotonin, and Spermine.

To gain an insight into the metabolic changes of MELAS, a targeted metabolomic analysis of 188 metabolites was carried out on cellular extracts from cybrid SHSY-5Y neuronal cells with different m.3243A > G mutation loads (i.e., 70%, 90%, and 98%), and compared to control cells. Unsupervised principal component analysis (PCA) of metabolomic data from parental cells (n = 10) and cells with the MELAS mutation with different mutation loads (n = 30) revealed a clear distinction between mutant and non-mutant cells (Figure 1A,B), as well as between cells with different mutation loads (Figure 1C), which validates the metabolomic analysis.

Compared to control cells, mutant cells with 70 to 98% mutation loads revealed a metabolic signature characterised by significantly lower levels of six acylcarnitines (i.e., C0, C2, C4, C16, C18, C18:1) and 10 amino acids (i.e., alanine, glutamine, serine, lysine, proline, glycine, serotonin, spermine, arginine, and taurine) (Figure 1D). Interestingly, arginine and taurine, two key amino acids already known to be involved in the pathophysiology of MELAS were significantly reduced in mutant cells. In addition, several phospholipids, lysophospholipids, and sphingomyelins were significantly increased in mutant cells (Figure 1D). Interestingly, the comparison of the metabolic signature of cells with the MELAS mutation with different mutation loads identified 10 amino acids (i.e., phenylalanine, tyrosine, valine, tryptophan, isoleucine, leucine, threonine, histidine, methionine, and alpha-aminoadipic acid), concentrations of which were significantly decreased between the 70% and 90% mutant cells, compared to 98% mutant cells (Figure 1E). Conversely, 14 metabolites, including 6 phosphocholines and lysophosphocholines, 8 amino acids (i.e., ornithine, acetyl ornithine, trans-4-hydroxyproline, aspartic acid, alanine, glutamine, glutamate, and DOPA) were significantly increased in 98% mutant cells, compared to lower mutation loads (Figure 1E), thus pointing to highly specific changes in amino acid and biogenic amine metabolite concentrations, according to the m.3243A > G heteroplasmy level.

### 3.2. The Level of Intracellular Glutamate Correlates Positively with the Heteroplasmy Level and Negatively with Mitochondrial Complex I Activity

**Figure 2:**
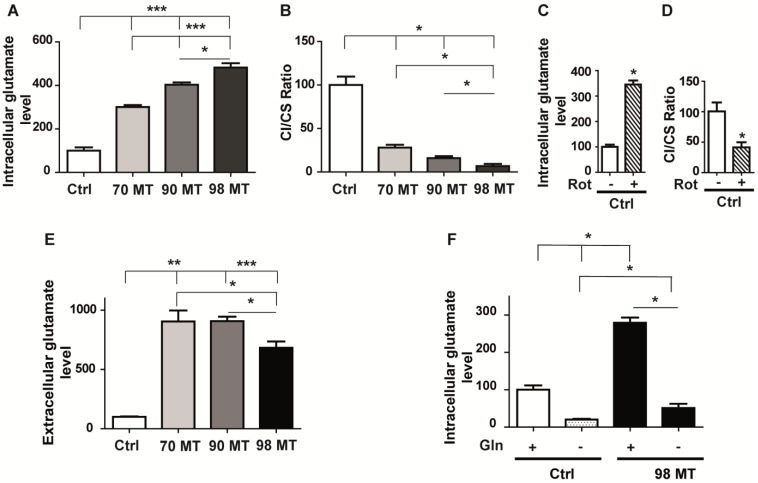
Glutamate concentration is correlated with complex I deficiency in MELAS cells. (**A**) Intracellular levels of glutamate in control (Ctrl) and mutant (MT) cells (70%, 90%, and 98%). (**B**) Biochemical assessment of mitochondrial complex I activity in Ctrl and MT cells (70%, 90%, and 98%). (**C**) Intracellular level of glutamate in Ctrl cells treated for 15 h with rotenone (200 nM) or a vehicle. (**D**) Biochemical assessment of mitochondrial complex I activity in Ctrl cells treated for 15 h with rotenone or a vehicle. (**E**) Extracellular glutamate levels. Ctrl: control cells and MT cells carrying different mutation loads (70%, 90%, and 98%). (**F**) Intracellular glutamate levels in Ctrl and 98% MT cells with (+) or without (−) the addition of glutamine. Results are presented as the mean ± SEM relative to Ctrl cells of at least 4 independent experiments. Statistical differences between MT and Ctrl cells are indicated with an asterisk (* *p* < 0.05; ** *p* < 0.01; *** *p* < 0.001).

Quantification of glutamate showed higher intracellular glutamate concentrations in cells with the MELAS mutation, reaching a four-fold increase in cells with a 98% mutation load compared to control cells (Figure 2A). Furthermore, the intracellular glutamate concentration was positively correlated with the heteroplasmy level in mutant cells (Figure 2A) and was inversely proportional to the complex I activity related to mitochondrial citrate synthase (CS) activity (Figure 2B). Likewise, the intracellular glutamate concentration was increased by 55% in 143B osteosarcoma cybrid cells with the m.3243A > G mutation and a 98% mutation load, compared to the 143B control cybrid cells (Figure S1A).

Next, we investigated the relationship between the level of glutamate and the activity of complex I by exposing control cells to rotenone, a complex I inhibitor, for 15 h. This experiment showed a 58.5% reduction in complex I activity compared to untreated cells, which was associated with a 245% increase in intracellular glutamate concentration, compared to untreated cells (Figure 2C,D). This suggests that the activity of mitochondrial complex I regulate the intracellular glutamate concentration.

In addition, the level of extracellular glutamate was also significantly increased in mutant cells of various mutation loads, compared to control cells (Figure 2E). Nevertheless, the extracellular glutamate concentration was lower in the 98% mutant cells compared to the 70% and 90% mutant cells (Figure 2E). 

Since glutamine is a precursor for the biosynthesis of glutamate, we next monitored the intracellular glutamate level in cells cultured with or without glutamine. We found a reduction in intracellular glutamate in both the control and 98% mutant cells grown in a glutamine-free medium, suggesting that most of the intracellular glutamate is derived from glutamine (Figure 2F). 

### 3.3. Transcriptomic Signature of MELAS in 98% Mutant Cells Shows Upregulation of the Glutamate and Glutamine Metabolic Pathways 

**Figure 3:**
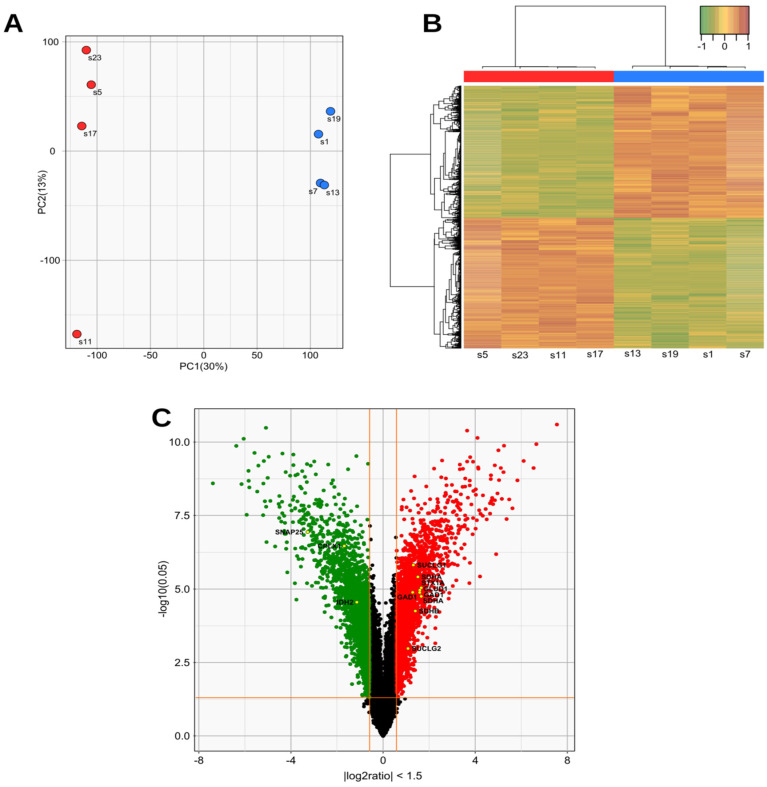
Gene expression profiling of mutant cells. (**A**) Principal component analysis (PCA) and unsupervised clustering of MT (red) vs Ctrl cells (blue). (**B**) Heatmap diagram of two-way hierarchical clustering analysis of the 4943 probes, showing different expression levels with a *p*-value ≤ 0.05 and abs (FC) ≥ 1.5. Red and green colours represent an expression level above or lower than the mean, respectively. The X-axis represents samples with, from the left to the right, control cells compared to 98% MT cells (n = 4) while the Y-axis represents Illumina probes. (**C**) Volcano plot representation of the differentially expressed genes in a pairwise comparison of control vs 98% MT cells. The significant cut-off was set at a *p*-value ≤ 0.05 and abs (FC) ≥ 1.5. Differentially expressed genes annotated as glutamate-glutamine metabolism, GABA, and TCA cycle in the REACTOME pathway database (see Table 1) are labelled with their corresponding gene symbols.

To gain further insights into the transcriptional regulation, gene expression profiling was carried out in the 98% mutant cells and compared to control cells. PCA and unsupervised clustering showed a clear difference between the 98% mutation load and control cells (Figure 3A). A comparison of the gene expression profile showed a total of 2046 differentially expressed genes with a *p*-value ≤ 0.05 (fold change ≥ 1.5) (Table S1). In the 98% mutant cells, 48% of genes were upregulated and 52% downregulated.

Analysis of these genes using the REACTOME pathway database predicted that a total of 955 metabolic pathways were involved in the process, with a *p*-value ≤ 0.05 (Table S2). The three main gene clusters involved were: (i) the glutamate and glutamine metabolism (R-HSA-8964539), (ii) the gamma-aminobutyric acid (GABA) synthesis, release, reuptake, and degradation; (R-HSA-888590) and (iii) the Tricarboxylic Acid Cycle (TCA) cycle (R-HSA-71403) (Table 1). This is in accordance with the glutamate and mitochondrial dysregulation revealed by our metabolomic analysis. The expression of a large number of genes involved in the glutamate and GABA pathways were significantly altered (Figure 3B, C). The most upregulated genes were two key genes of the glutamate pathway, namely *GLUD1* and *GAD1,* which encode glutamate dehydrogenase 1 (GDH) and glutamate decarboxylase 1 (GAD1) respectively. GDH converts L-glutamate into alpha-ketoglutarate (αKG), a key step in glutamine anaplerosis, whereas GAD1 converts L-glutamate into GABA.

In addition, in 98% mutant cells, several genes involved in the TCA cycle were also upregulated, including those which encode citrate synthase and isocitrate dehydrogenase, as well as most genes that encode succinate dehydrogenase complex and genes belonging to the succinate-CoA ligase family. It should be noted, however, that *ME3*, encoding the mitochondrial malic enzyme 3 which catalyzes the decarboxylation of malate to pyruvate was found to be downregulated in mutant cells (Table 1).

### 3.4. Exposure of Cells with the MELAS Mutation to Ketone Body (KB) Restores the Intracellular Glutamate Level, Improves Mitochondrial Dynamics, and Respiratory Chain Activity

We have previously shown that the mitochondrial function of cells with the MELAS mutation was improved by the switch from glycolysis to fatty acid β-oxidation induced by the exposure to KB combining acetoacetate and β-D-hydroxybutyrate [21]. We, therefore, investigated the effect on glutamate levels of exposure to KB for 48 h on the 98% mutation load cells. 

**Figure 4:**
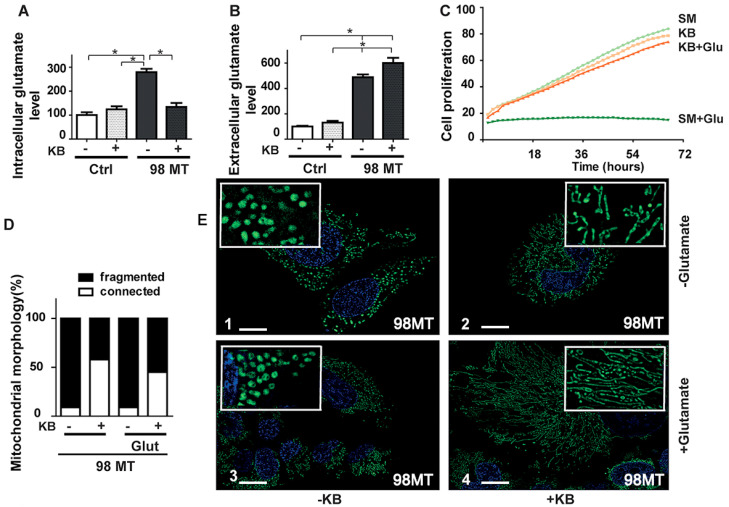
Treatment with ketone bodies restores a normal intracellular glutamate concentration and improves the mitochondrial network in MELAS cells. (**A**) Intracellular and (**B**) extracellular glutamate levels in Ctrl and 98% MT cells treated for 48 h with (+) or without (−) KB. Results are from at least four independent experiments, expressed as the mean ± SEM relative to Ctrl cells. (* *p* < 0.05). (**C**) Cell growth of 98% MT cells cultured in a standard medium (SM, light green curve), or exposed to KB (orange curve), or with 50 µM glutamate (Glu) and KB (red curve) or a standard medium (SM + Glu, green curve). (**D**) Mitochondrial morphology, and percentages of fragmented (black) or connected (white) mitochondria in 98% MT cells with (+) or without (−) KB, and with or without glutamate (30 µM). (**E**) Representative images showing the MitoTracker (green fluorescence) and Hoechst (blue fluorescence) staining of 98% MT cells, incubated for 24 h in E-1: standard medium, E-2: with KB, E-3: with glutamate (Glu), and E-4: with Glu and KB. Scale Bar: 10 um.

The exposure to KB resulted in a 48% reduction in the intracellular glutamate level in the 98% mutant cells, whereas the extracellular glutamate concentration remained unchanged, and no variation of intra- or extracellular glutamate levels was observed in the control cells (Figure 4A,B). Similar results on intracellular glutamate levels were obtained with the 143B cybrid mutant cells carrying a 98% mutation load of the MELAS mutation, whereas KB exposure significantly increased intracellular glutamate levels in 143B control cells (Figure S1A).

As glutamate has been shown to be toxic to neuronal activity and cell growth [34], we evaluated the protective role of KB, after exposing 98% mutant cells to high glutamate concentrations (30–50 µM range). After incubation in a standard medium (SM) or KB, cell proliferation of the 98% mutant cells was measured in the presence or absence of glutamate. Cellular growth of the 98% mutant cells was fully inhibited in the presence of glutamate in SM whereas glutamate did not affect their proliferation in the presence of KB (Figure 4C). We further observed a close inverse correlation between growth rates and intracellular glutamate concentrations.

In addition to these effects on cell growth, exposure to KB also inhibited cell death induced after 15 h incubation with 30 μM of glutamate (Figure S1B).

We next investigated the toxicity of a high intracellular glutamate concentration on the mitochondrial network dynamics. While the mitochondrial network was highly connected in control cells, it was fragmented in the 98% mutation load untreated cells (Figure 4D,E). Interestingly, KB treatment restored mitochondrial fusion and network elongation in cells with the MELAS mutation (Figure 4D,E). Conversely, control cells exposed to a high glutamate concentration presented mitochondrial swelling and fragmentation, while further addition of KB restored a connected network, demonstrating that treatment with KB has a protective effect against glutamate toxicity and restores normal mitochondrial dynamics (Figure S1D).

### 3.5. Mitochondrial Respiration and Enzyme Activities Are Restored by KB Exposure in Cells with the MELAS Mutation

**Figure 5:**
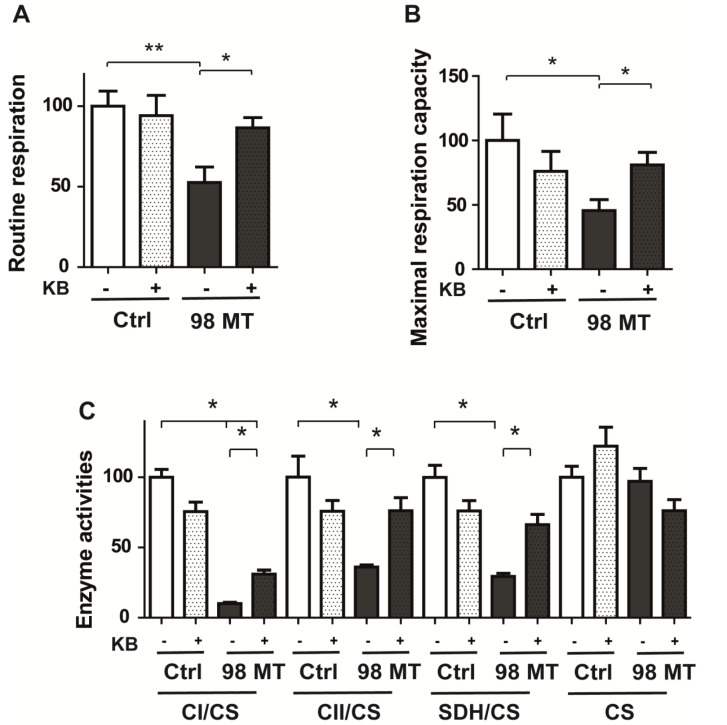
Treatment with ketone bodies improves mitochondrial respiration and enzyme activities in MELAS cells. (**A**) Oxygraphic measurements of routine (**B**) and maximal respiration capacity in Ctrl and 98% MT cells, treated with or without KB for 48 h. (**C**) Enzyme activities of mitochondrial complex I, II, and SDH, relative to citrate synthase (CS) in control and 98% MT cells, treated for 48 h with or without KB. Results are presented as the mean ± SEM, relative to Ctrl cells, of at least 4 independent experiments. Statistical differences between 98% MT and Ctrl cells are indicated with an asterisk (* *p* < 0.05; ** *p* < 0.01).

Treatment with KB in cells with the MELAS mutation resulted in a 30% increase in the routine respiration capacity and a 56% increase in the maximal respiration capacity compared to untreated 98% mutant cells, while mitochondrial respiration of treated or untreated control cells was unchanged (Figure 5A,B). This was paralleled with a significant two-fold increase in mitochondrial enzyme activities of complex I and II, related to citrate synthase, in cells treated with KB for 48 h compared to untreated cells (Figure 5C). 

### 3.6. The m.3243A > G MELAS Variant Affects the Glutamate/GABA Pathways

**Figure 6:**
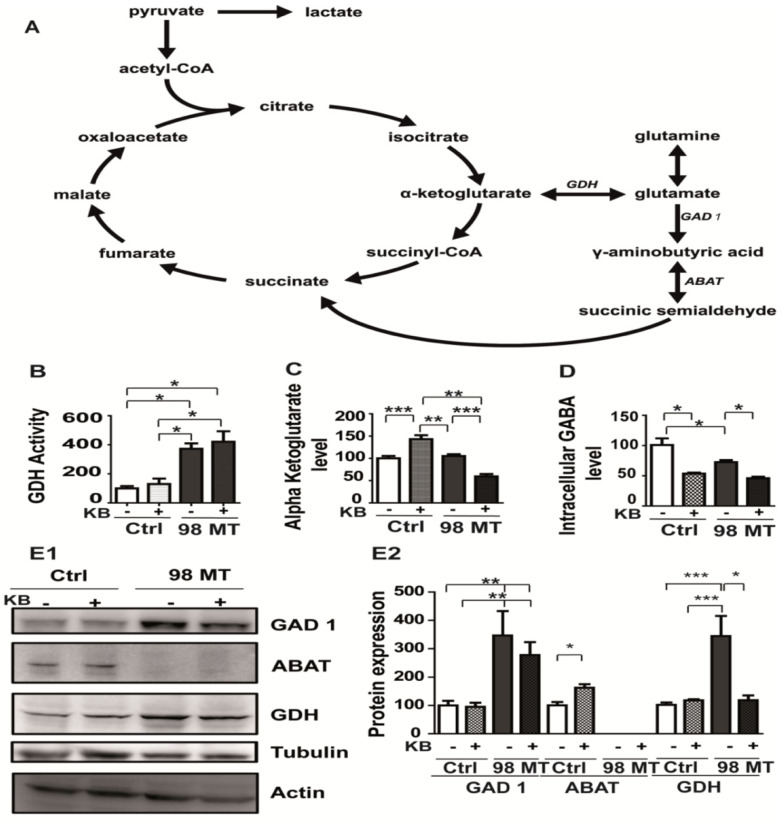
The Glutamate and GABA metabolic pathways are altered in MELAS cells. (**A**) Graphical representation of the glutamate pathway and TCA cycle. GAD1 (glutamate decarboxylase), ABAT (4 aminobutyrate transaminase), and GDH (glutamate dehydrogenase). (**B**) Measurement of GDH activity, (**C**) intracellular levels of αKG concentration, and (**D**) intracellular levels of GABA in Ctrl and 98% MT cells, exposed for 48 h with (+) or without (−) KB. (**E1**) Western blots showing GAD1, ABAT, and GDH expression profiles in Ctrl and 98% MT cells, treated for 48 h with (+) or without (−) KB. (**E2**) Quantification of GAD1, ABAT, and GDH relative expression related to tubulin and actin in Ctrl and 98% MT cells treated with (+) or without (−) KB. Results are presented as the mean ± SEM, relative to Ctrl cells of at least 4 independent experiments. Statistical differences between 98% MT and Ctrl cells are indicated with an asterisk (* *p* < 0.05; ** *p* < 0.01; *** *p* < 0.001).

Based on our gene expression results, the main enzymes involved in glutamate metabolism pathways, downstream of the transformation of glutamine to glutamate by the glutaminase, were investigated. The first pathway consists of generating αKG from glutamate by means of glutamate dehydrogenase (GDH) [35]) (Figure 6A). The second pathway generates succinate by the successive involvement of glutamate decarboxylase (GAD1), catalysing the decarboxylation of glutamate *to* GABA, the 4-aminobutyrate aminotransferase (ABAT) leading to succinic semialdehyde (SSA), and then the succinate-semialdehyde dehydrogenase (SSADH) leading to succinate, the substrate of complex II (Figure 6A). Importantly, defects in these enzymes are responsible for severe neurometabolic disorders, seizures, intellectual disability, and generalised dystonia [36,37,38].

Gene expression profiling of 98% mutant cells (Figure 3B,C) highlighted the overexpression of GLUD1, which encodes GDH (Figure 6A and Table 1), prompting us to monitor the conversion of glutamate into αKG by the GDH enzyme. A four-fold increase in GDH activity in 98% mutant cells was observed (Figure 6B), paralleling a concomitant overexpression of the protein (Figure 6(E1,E2)), while the αKG level remained unchanged in both the control and 98% mutant cells (Figure 6C).

Interestingly, GDH enzyme activity was unchanged by KB exposure, whereas the concentration of αKG, as measured by mass spectrometry, was significantly reduced (71%) in mutant cells exposed to KB (Figure 6C). This suggests that KB stimulates the mitochondrial anaplerotic energy metabolism in mutant cells, providing alternative substrates capable of replenishing the intermediates of the TCA cycle [39]. We also observed that the αKG concentration was also increased in KB supplemented control cells (Figure 6C). In addition, GABA levels were reduced in both mutant and control cells exposed to KB by 63% and 48%, respectively (Figure 6D). 

We further assessed the protein expression level of GAD1, ABAT, and GDH from the glutamate pathway which revealed that GAD1 and GDH were significantly increased (two-fold) in 98% mutant cells. A normal GDH expression level was restored by KB exposure in the 98% mutant cells (Figure 6(E1,E2)). Surprisingly, we found that ABAT mRNA expression was completely inhibited in mutant cells and the protein level was undetectable in Western blotting (Figure 6(E1,E2) and Figure S2). 

The expression level of ABAT protein was unchanged after treatment with KB in mutant cells (Figure 6(E1,E2)). In addition, we confirmed a drastic increase in GAD1 mRNA in the 98% mutation load compared to the control cells (Figure S2), in line with the GAD1 overexpression seen in the transcriptomic analysis (Table 1, Figure 6(E1,E2)).

### 3.7. Dysfunction of the Mitochondrial TCA Cycle in Cells with the MELAS Mutation Is Alleviated by KB Exposure 

**Figure 7:**
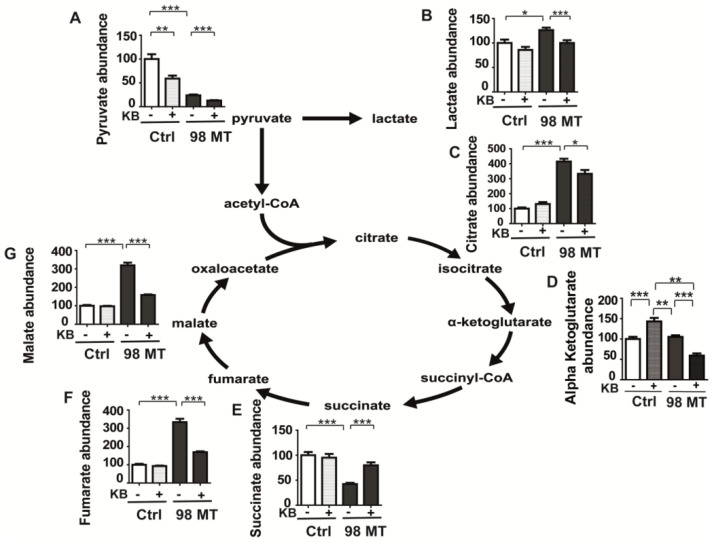
TCA cycle dysfunction in MELAS cells is alleviated by ketone bodies. (**A**) Pyruvate, (**B**) lactate, (**C**) citrate, (**D**) αKG, (**E**) succinate, (**F**) fumarate, and (**G**) malate levels in Ctrl and 98% MT cells treated for 48 h with (+, dotted line) or without (−, colour bar) ketone bodies (KB). Results are presented as the mean ± SEM relative to Ctrl cells of at least 4 independent experiments. Statistical differences between 98% MT and Ctrl cells are indicated with an asterisk (* *p* < 0.05; ** *p* < 0.01; *** *p* < 0.001).

The measurements of TCA intermediates by mass spectrometry showed that the pyruvate concentration was reduced by 76% in cells with the MELAS mutation compared to control cells since most of the pyruvate produced during glycolysis was converted into lactate (Figure 7A,B), as expected in mitochondrial dysfunction [24]. Furthermore, citrate concentration was four times higher in the 98% mutation load than in control cells, representing one of the blockade points of the TCA cycle in 98% mutant cells (Figure 7C), while succinate concentration was reduced by more than half in 98% mutant cells compared to control cells (Figure 7E). In addition, malate and fumarate concentrations were increased by 319% and 333%, respectively, revealing two additional blocking steps in the TCA cycle related to the reduction in the oxidation of different substrates by the NAD+/NADH ratio (Figure 7F,G). The level of αKG remained unchanged between the control and 98% mutant cells, suggesting that αKG was converted to glutamate via the GDH bidirectional enzyme, a process also responsible for the glutamate accumulation in mutant cells (Figure 6C and Figure 7D).

Interestingly, KB treatment resulted in both a 88% reduction in pyruvate concentration (Figure 7A) as well as a reduction in lactate production in KB-treated 98% mutant cells, compared to untreated cells, suggesting that glycolysis was reduced (Figure 7B). Importantly, KB treatment allowed the TCA cycle to proceed in a more physiological manner, unlocking the various NADH-dependent TCA cycle blockages, like citrate, malate, fumarate, and αKG concentrations were significantly lowered by treatment with KB (Figure 7C,D,F,G), while succinate concentration was increased (Figure 7E). In KB-treated control cells, a 47% reduction of pyruvate was also observed and a 50% increase in αKG, without any other metabolic changes (Figure 7A–D).

### 3.8. The Glutamate Pathway Is Altered in the Brain Tissue of a Patient with MELAS

**Figure 8:**
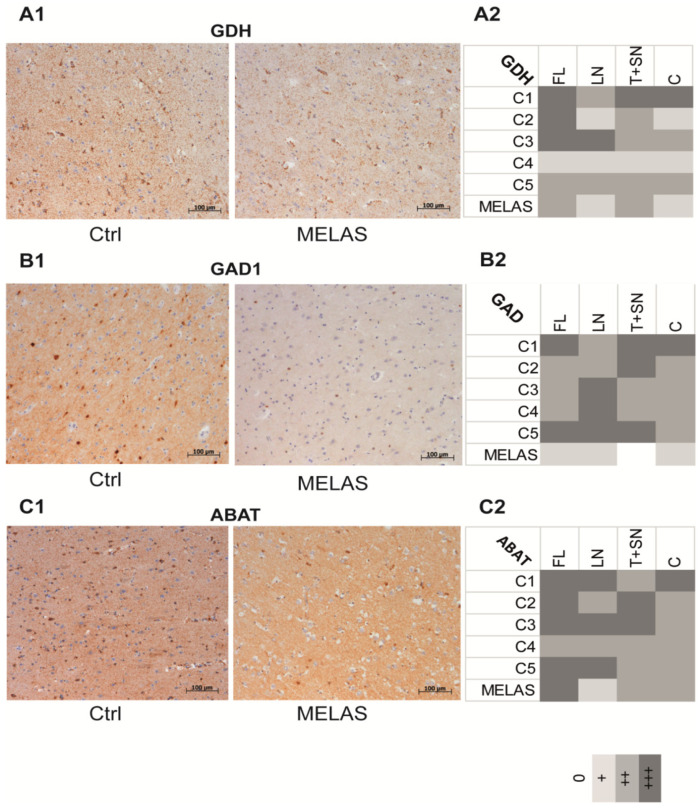
The glutamate pathway is altered in the brain tissue of a patient with MELAS. Immunohistochemical analysis of paraffin-embedded human frontal brain tissue labelled with GDH (**A1**,**A2**), GAD1 (**B1**,**B2**), and ABAT (**C1**,**C2**) antibodies, from Ctrl individuals (left panel) and a patient with MELAS (right panel). Immunohistochemical staining intensities of GDH (**A2**), GAD1 (**B2**), and ABAT (**C2**) were examined microscopically and scored semi-quantitatively as part of two independent analyses on five Ctrl individuals and one patient with MELAS as follows: 0 = absent, + = mild, ++ = moderate, and +++ = intense. FL: frontal lobe; LN: lentiform nucleus; T+SN: thalamus + subthalamic nucleus, C: cerebellum.

Mass spectrometry was performed on post-mortem brain tissue obtained from a 32-year-old woman who died of MELAS caused by the m.3243A > G variant and compared to control brains. Higher concentrations of amino acids (Table 2), including glutamine, glutamate, alanine, proline, arginine, histidine, ornithine, isoleucine, methionine, threonine, and valine were found in the brain with the MELAS mutation compared to control brains. Most of these amino acids contribute to the glutamate pathway, thus their increase strongly suggests that glutamate catabolism was also impaired in the brain tissues with the MELAS mutation. Similar to cells with the MELAS mutation, the concentration of GABA was also reduced in the brain, while immunohistochemical studies revealed a reduction in GDH, GAD1, and ABAT staining in the frontal lobe, lentiform nucleus, thalamus plus subthalamic nucleus, and cerebellum areas of the brain when compared to five control brains (Figure 8A–C). 

## 4. Discussion

Clinical presentations of mitochondrial disorders caused by pathogenic mtDNA variants are highly heterogeneous due to the peculiarities of the mitochondrial genome [1]. Moreover, the development of mitochondrial medicine is also greatly impaired by the lack of biomarkers to monitor the disease outcome and by the absence of efficient therapies which can target mitochondria [1]. To tackle these restrictions, substantial progress has been made in recent years using OMICS technologies, providing novel insights into disease mechanisms and opportunities to develop therapeutic routes [40,41,42]. 

The m.3243A > G variant is considered to be one of the most common mtDNA mutations and the consequences of clinical heterogeneity and mutation load are still not well understood [37]. This particular variant is responsible for MELAS syndrome or Maternal Inherited Diabetes and Deafness (MIDD) as well as other related clinical disorders [6]. Here, we have applied a strategy based on an integrated approach using emerging technologies with the combination of targeted metabolomics and transcriptomics to assess the consequences of the m.3243A > G mutation heteroplasmy in a neuronal cell model of MELAS. The main goal was to identify a metabolic signature and relevant pathways, and ultimately to monitor the efficacy of nutritional-based therapy with the use of ketone bodies.

*A multi-omic signature of cells with the MELAS mutation.* A specific biochemical signature revealing key biomarkers of the MELAS syndrome was identified using targeted metabolomics based on high-performance liquid chromatography coupled with tandem mass spectrometry and latent variable-based statistical methods. The quantification of 188 metabolites in neuronal cybrid cells with the MELAS mutation confirmed the significant reduction in arginine and taurine, a result that has already been identified with MELAS syndrome, further supporting the therapeutic use of these two key amino acids in patients with MELAS with the goal of reducing stroke-like episodes [16,38,39]. 

In addition, metabolomic results evidenced a significant increase in L-DOPA, alanine, glutamate, and glutamine. L-DOPA accumulation was shown to contribute to mitochondrial dysfunction in an in vitro cellular model of Parkinson’s disease [43] whereas increased alanine concentration has been considered to be a hallmark of mitochondrial disorders [1]. However, the most unexpected result of the metabolomic analysis was the drastic increase in glutamate and glutamine in cells with the MELAS mutation compared to controls.

**Figure 9:**
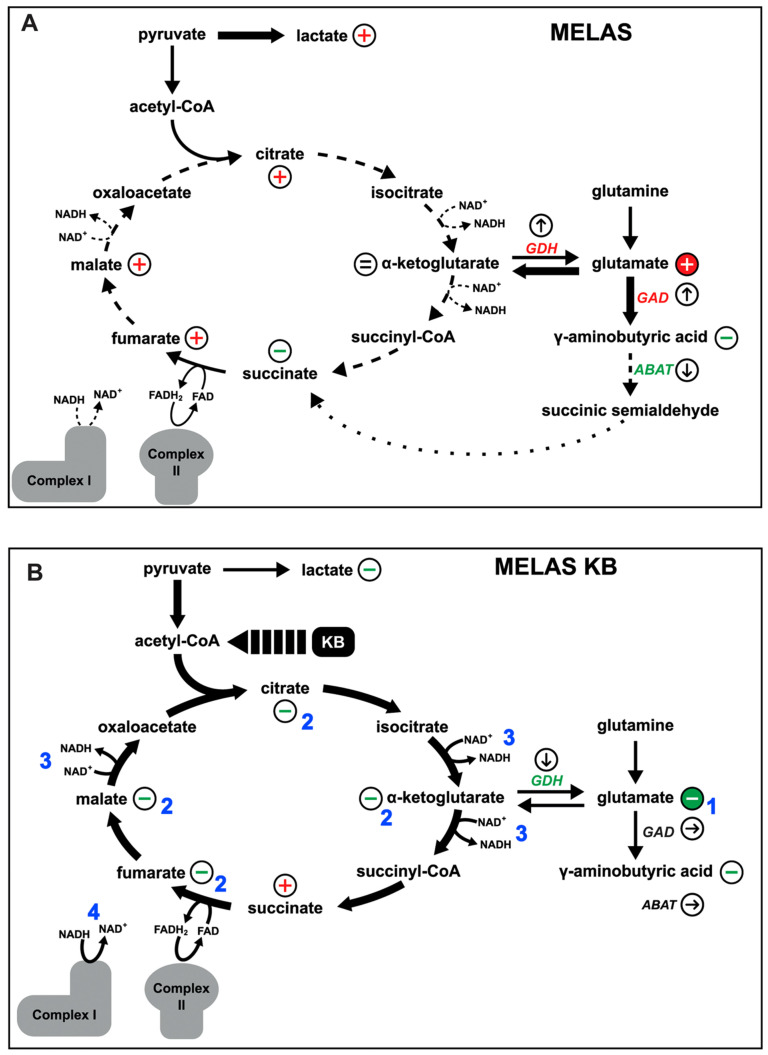
Graphical representation of metabolic pathways of MELAS cells (**A**) untreated (**B**) or treated with ketone bodies (KB). +: metabolite increase. –: metabolite reduction, = metabolite unchanged. ↑: increased gene expression. ↓: decreased gene expression. Metabolic consequences of KB treatment on mitochondrial metabolism are summarised in 4 main steps: 1. significant reduction of glutamate concentration; 2. reduction of the accumulation of TCA intermediates restoring the physiological function of the TCA cycle; 3. re-equilibration of the redox/NADH balance; 4 improving complex I enzyme activity.

*Glutamate is a key marker of MELAS pathophysiology*. Glutamate, whose dysregulation is frequently encountered in neurological disorders, is a non-essential amino acid crucial for neurotransmission in the central nervous system [44,45] and glutamate has also a key role to fuel αKG into the mitochondrial TCA (Figure 9A). Our multi-omic approach highlighted the metabolic dysregulation of the glutamate pathway and established firm correlations between intracellular glutamate level, mitochondrial complex I inhibition, and the m.3243G>A heteroplasmy level in cells with the MELAS mutation. These observations were confirmed by the transcriptomic data which revealed the glutamate metabolism ontology to be one of the most affected in cells with the MELAS mutation, together with the GABA and TCA cycle pathways. Among these gene clusters, those encoding the key enzymes GAD1 and GDH involved in the glutamate pathway were highly upregulated in mutant cells. This is in line with what we have previously observed in 143B cybrid cells with the MELAS mutation, further providing evidence for the correlation of the transcriptional reprogramming with the heteroplasmy level [46].

Extracellular glutamate concentrations were also significantly elevated in the supernatant of cybrid neuronal cells with the MELAS mutation, especially those carrying the 70% and 90% mutation loads, while it was also increased, but to a lower extent, in the 98% mutant cells. This could possibly be explained by an inhibition of glutamate transport in this latter condition, as previously reported [47]. 

*Intermediate metabolites of the TCA cycle are increased in cells with the MELAS mutation.* Our metabolomic results demonstrated an accumulation of TCA cycle intermediates such as citrate, malate, and fumarate, evidencing the blockade of the TCA cycle in cells with the MELAS mutation. Importantly, these substrates were related to NADH oxidation, which is dependent on mitochondrial complex I activity. These results are fully in line with those of a recent clinical study performed in a large cohort of patients with the m.3243A > G variant which evidenced the accumulation in plasma of intermediate metabolites of the TCA cycle which was linked to NAD+-dependent biochemical reactions [42]. The identification of those metabolites related to NADH redox unbalance reflects the disease severity and may be used as biomarkers to follow candidate therapies aimed at reducing NADH reductive stress [48,49]. In addition, in the context of homoplasmic mtDNA mutations, it has been shown that glutamine, the precursor of glutamate, was used within the TCA cycle, leading to the increased synthesis of aspartate [39]. Thus, increased amino acid levels, including glutamate levels, are consistent with a compensatory response to the primary MELAS OXPHOS defect [39,50]. A recent study aimed to assess glutamate and glutamine levels in plasma and cerebrospinal fluid in MELAS patients showed an elevated glutamate level in the CSF but not in the plasma [51] and in contrast, the CSF glutamine level was lower than controls [51]. These data raise the question of glutamate-glutamine balance in the pathophysiology of MELAS.

*Mitochondrial dysfunction is alleviated by Ketone Body exposure*. To monitor if the toxic impact of a high glutamate concentration could be reduced, cells with the MELAS mutation were exposed to treatment with KB which led to a switch from a glycolytic metabolism to mitochondrial fatty acid oxidation. Importantly, cells with the MELAS mutation exposed to KB unlocked the NADH-dependent TCA cycle blockade, restoring a normal TCA cycle function and significantly increasing oxidative metabolism (Figure 9B). Indeed, analysis of the TCA cycle revealed a reduction in citrate, malate, and fumarate metabolites, all dependent on NADH oxidation, thus restoring physiological TCA cycle function. These results suggest that a ketogenic diet could be beneficial in treating the MELAS disease, as already proposed for other mitochondrial diseases and for drug-resistant epilepsy [20,22,23]. Further in vitro test results have been recently obtained in our laboratory which demonstrates that KB exposure over a period of 4 weeks significantly alleviates mitochondrial dysfunction in cells with the MELAS mutation by improving complex I assembly and activity [21]. KB has also been shown to reduce the percentage of deleted mtDNA molecules in cells with large-scale heteroplasmic deletions after only 5 days of exposure [52]. Other studies have also demonstrated that treatment of SH-SY5Y parental cells or complex I deficient cells with decanoic acid, a component of the medium chain triglyceride form of the ketogenic diet, improve mitochondrial functions [53,54].

In addition, KB exposure reduced the accumulation of glutamate in cells with the MELAS mutation (Figure 9B), while increasing αKG concentration, suggesting that this latter metabolite is an important marker of anaplerotic energy metabolism [39]. αKG also plays a crucial role in the pathophysiology of other mitochondrial disorders and in age-related neurodegenerative diseases, which is most probably related to the reduction of the αKG dehydrogenase complex, as evidenced in age-related neurodegenerative disorders [55]. Perturbations of the glutamine/glutamate/αKG metabolic axis were also found in the *Ndufs4* KO mouse model of Leigh syndrome while being rescued by mTOR inhibition with rapamycin [56]. This latter study also highlighted that αKG is a complex I substrate that supports oxidative phosphorylation [56]. 

*The level of GABA is significantly reduced in neuronal cells with the MELAS mutation as well as in post-mortem brain tissue.* In parallel with the accumulation of other amino acids such as glutamate, we observed a significant reduction in *GABA* in neuronal cells with the MELAS mutation without changing the expression level of ABAT that was confirmed in brain tissue obtained from a patient with MELAS. Mitochondrial dysfunction affects the storage, release, or uptake mechanisms of neurotransmitters including glutamate and GABA [57]. This is in line with the high prevalence of neuropsychiatric symptoms seen in patients with MELAS [58]. The uptake of glutamate and GABA in synaptosomal preparations from mouse brains was also decreased by rotenone, dependent on the dose [59]. Interestingly, it was recently demonstrated that the regulation of GABA availability and signalling was strongly connected with mitochondrial alterations which in turn modulates neuronal communication and social behaviour [60]. Of note, ABAT variants were also responsible for encephalomyopathies with a neurometabolic disorder of GABA degradation resulting in an elevated GABA in the brain as well as mtDNA depletion syndrome highlighting ABAT as an enzyme of dual function [61]. 

As shown in our study, the ketogenic diet appears to be a promising strategy for modulating mitochondrial function, and eventually mitochondrial biogenesis [21,62]. New imaging technologies such as magnetic resonance imaging and positron emission tomography may be used to monitor the disease outcome following key metabolic biomarkers during KB treatment [63]. In this respect, an open-label, non-randomised clinical trial based on treatment with bezafibrate, a molecule known to stimulate mitochondrial biogenesis, was recently evaluated on a limited number of patients carrying the m.3243A > G mutation [64]. The results revealed an alteration of glutamine metabolism with a significant increase in several amino acids including alanine and glutamate which is thought to be part of a compensatory response to the OXPHOS defect in the context of mtDNA mutation [40,48].

## 5. Conclusions

This work highlights the usefulness of a multi-omic strategy and significantly improves our understanding of the contribution of glutamate metabolism to the MELAS pathophysiology, promoting glutamate as a potential biomarker of severity for MELAS syndrome but also demonstrating the consequences of an NADH/ NAD^+^ redox imbalance. Metabolic interventions such as a ketogenic diet should be considered a promising therapeutic route to help reduce glutamate toxicity, alleviating the blockades of the TCA cycle by reducing NADH reductive stress due to complex I deficiency in MELAS syndrome. 

## Figures and Tables

**Table 1:** Functional classification analysis of repressed or overexpressed genes in MELAS cells according to the REACTOME pathway database.

Pathway ID	Pathway Description	Upregulated Genes	Downregulated Genes	Unchanged Genes
R-HSA-8964539	Glutamate and glutamine metabolism	* **ALDH18A1, GLUD1, GOT2, KYAT1, OAT, RIMKLB** *	* **PYCR1** *	*GLS, GLS2, GLUD2, GLUL, PYCR2, PYCR3, RIMKLA*
R-HSA-888590	GABA synthesis, release, reuptake and degradation	* **GAD1, HSPA8, SLC6A1, STX1A, VAMP2** *	* **ALDH5A1, CPLX1, SNAP25, SYT1** *	*ABAT, DNAJC5, GAD2* *RAB3A, RIMS1,SLC32A1* *SLC6A11, SLC6A12, SLC6A13, STXBP1*
R-HSA-71403	TCA cycle	* **CS, DLST, IDH3A, IDH3B, IDH3G, OGDH, SDHA, SDHB** * * **SDHD, SUCLA2, SUCLG1** * * **SUCLG2** *	* **FAHD1, IDH2, ME3, NNT, SDHC** *	*ACO2, DLD, FH, MDH2, ME2*

The three key clusters (glutamate, GABA, and TCA cycle) from the reactome are shown. Genes belonging to “glutamate and glutamine metabolism”, “GABA synthesis, release, reuptake, and degradation” and “TCA cycle” were classified into three groups: upregulated genes with a *p*-value ≤ 0.05 and FC ≥ 1.3, down-regulated genes with a *p*-value ≤ 0.05 and FC ≤ −1.3 and unchanged gene expression if the *p*-value > 0.05 and/or abs (FC) < 1.3. Upregulated and downregulated genes from these pathways with a fold change over 1.5 are indicated in bold.

**Table 2:** Mass spectrometry quantification of amino acids in post-mortem brain tissue from Ctrls and a patient with MELAS.

Amino Acids	Ctrls	Patient with MELAS
Glutamate	239 ± 15.5	417
Glutamine	246.5 ± 0.7	356
Proline	29.7 ± 0.42	60.1
Arginine	21.8 ± 0.28	52.9
Histidine	16.35 ± 2.19	25.4
Ornithine	10.75 ± 0.07	9.9
Isoleucine	16.1 ± 1.7	40.5
Methionine	9.64 ± 1.64	25.5
Threonine	25.15 ± 1.62	44
Valine	25.9 ± 2.54	62.3
Alanine	99.25 ± 3.9	168
Aspartate	133 ± 9.89	108
GABA	93.25 ± 7.2	66.9

Controls: Ctrls; Ctrl values (n = 2) are expressed as the mean ± SEM.

## Data Availability

GEO; accession number: GSE165953 on the GEO NCBI site.

## Supplementary Materials

The following supporting information can be downloaded at: https://www.mdpi.com/article/10.3390/biomedicines10071665/s1, Figure S1: The Glutamate pathway is also affected in 143B osteosarcoma MELAS cybrid cells; Figure S2: Gene expression of *GAD1* and *ABAT* by quantitative PCR. Table S1 Gene expression profile and Table S2 metabolic pathways.
