# Supplementary material for: Glutamate-Induced Deregulation of Krebs Cycle in Mitochondrial Encephalopathy Lactic Acidosis Syndrome Stroke-Like Episodes (MELAS) Syndrome Is Alleviated by Ketone Body Exposure
Source: Biomedicines. 2022 Jul 11;10(7):1665. doi: 10.3390/biomedicines10071665 (PMC9312837; doi:10.3390/biomedicines10071665)
Supplement: Supplementary file 1 [file biomedicines-10-01665-s001.zip › biomedicines-1758191-supplementary-Figure.pdf]

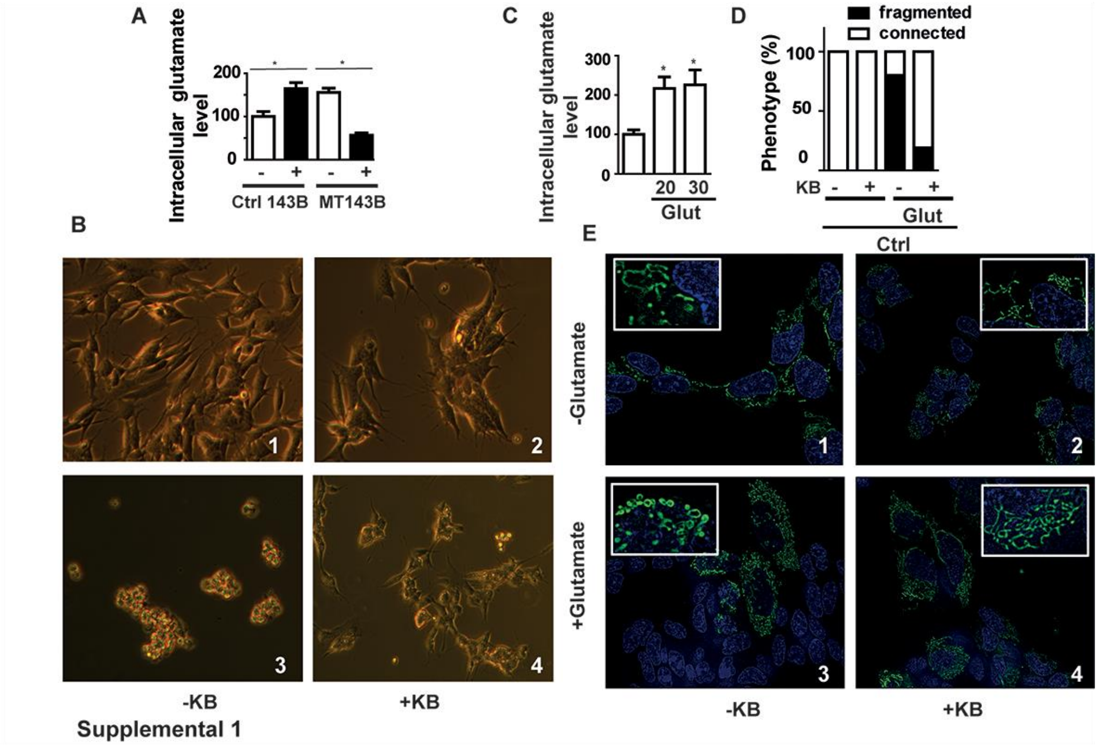

Figure S1. The Glutamate pathway is also affected in 143B osteosarcoma MELAS cybrid cells.

(A) Intracellular levels of glutamate in 143B osteosarcoma cybrid cells. Cybrid control (Ctrl) cells and MELAS 98% mutant (MT) cells. Results are presented as means ± SEM relative to Ctrl cells of at least 4 independent experiments. Statistical differences are indicated with an asterisk between 98% MT and Ctrl cells (*p<0.05).

(B) Glutamate impacts mitochondrial morphology in 143B cybrid Ctrl cells. Ctrl cells in standard medium (1), with ketone bodies (KB) (2), with 30mM glutamate, with KB + 30mM glutamate (4).

(C) Intracellular level of glutamate in Ctrl cybrid cells exposed for 15h to 20 and 30 mM glutamate.

(D) Glutamate impacts on mitochondrial network structure in control cells in the presence or absence of ketone bodies (KB). Results are presented as means ± SEM relative to Ctrl cells of at least 4 independent experiments. Statistical differences are indicated with an asterisk between 98% MT and Ctrl cells (*p<0.05; ** p<0.01; *** p<0.001). Representative images of cells stained with MitoTracker (green) and Hoescht-DNA staining (blue) incubated for 15h in standard medium (1), with KB (2), with 30mM of glutamate (3), or with KB + 30mM of glutamate (4).

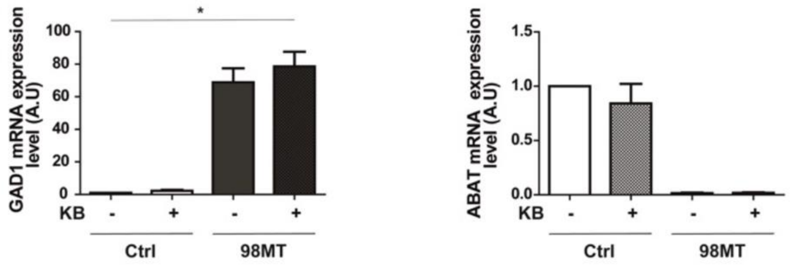

Figure S2. Gene expression of *GAD1* and *ABAT* by quantitative PCR
Gene Expression showing *GAD1, ABAT* expression levels in Ctrl and 98% MT cells, treated for 48h with (+) or without (-) KB.
